# Multi-Locus Molecular Findings in Pediatric-Onset Inherited Metabolic Disorders: An Eight-Year Series of Outsourced Genomic Testing in Taiwan

**DOI:** 10.3390/diagnostics16203356

**Published:** 2026-10-15

**Authors:** Chung-Lin Lee, Ya-Hui Chang, Chih-Kuang Chuang, Huei-Ching Chiu, Yuan-Rong Tu, Yun-Ting Lo, Jun-Yi Wu, Huang-Ying Huang, Hsiang-Yu Lin, Shuan-Pei Lin

**Affiliations:** 1 Department of Pediatrics, MacKay Memorial Hospital, Taipei 104217, Taiwan; clampcage@gmail.com (C.-L.L.); wish1001026@gmail.com (Y.-H.C.); g880a01@mmh.org.tw (H.-C.C.); 2 Institute of Clinical Medicine, National Yang-Ming Chiao-Tung University, Taipei 112304, Taiwan; 3 International Rare Disease Centre, MacKay Memorial Hospital, Taipei 104217, Taiwan; andy11tw.e347@mmh.org.tw (Y.-T.L.); wl01723138@gmail.com (J.-Y.W.); 4 Department of Medicine, Mackay Medical University, New Taipei City 252005, Taiwan; 5 Mackay Junior College of Medicine, Nursing and Management, New Taipei City 252005, Taiwan; 6 Division of Genetics and Metabolism, Department of Medical Research, MacKay Memorial Hospital, Taipei 104217, Taiwan; mmhcck@gmail.com (C.-K.C.); likemaruko@hotmail.com (Y.-R.T.); 7 College of Medicine, Fu-Jen Catholic University, New Taipei City 242062, Taiwan; 8 Department of Nursing, MacKay Memorial Hospital, Taipei 104217, Taiwan; hhy6543@mmh.org.tw; 9 Department of Medical Research, China Medical University Hospital, Taichung 404327, Taiwan; 10 Department of Infant and Child Care, National Taipei University of Nursing and Health Sciences, Taipei 112303, Taiwan

**Keywords:** whole-exome sequencing, whole-genome sequencing, inborn errors of metabolism, lysosomal storage disease, multi-locus diagnosis, blended phenotype, diagnostic odyssey, Taiwan

## Abstract

**Background/Objectives**: Outsourced genomic testing can identify additional findings in patients with inherited metabolic disorders (IEMs), including lysosomal storage diseases. We examined these findings in a selected Taiwanese series. **Methods**: We retrospectively reviewed 13 patients referred during 2018–2025 with a reported or suspected metabolic diagnosis. Whole-exome sequencing (WES) was initially used mainly after inconclusive testing or for incompletely explained phenotypes and was used more broadly later. The original registry also records whole-genome sequencing (WGS) for two included patients. We distinguished reported gene-level findings from diagnoses supported by pathogenic or likely pathogenic (P/LP) variants at each locus. **Results**: The 13 patients had 18 reported gene-level findings comprising 27 variants. Five met the inclusive multi-locus definition (38.5%; exact 95% confidence interval, 13.9–68.4%). The proportion was 30.8% after excluding candidate gene–disease associations and 7.7% when P/LP variants in the expected zygosity were required at both loci using the later case-summary classifications. Several classifications differed between the original registry and later summaries. These estimates are not directly comparable with the published 4.9% benchmark because ascertainment and diagnostic definitions differ. Enzyme replacement therapy was documented in three patients, including one treated before WES. **Conclusions**: Unexplained features may warrant evaluation for an additional diagnosis. The selected sample and differing levels of diagnostic certainty limit estimates of confirmed multi-locus disease.

## 1. Introduction

Whole-exome sequencing (WES) is now part of routine evaluation for children with suspected Mendelian disorders. The American College of Medical Genetics and Genomics recommends considering exome or genome sequencing as a first- or second-tier test for pediatric patients with congenital anomalies, developmental delay, or intellectual disability [1]. A large study of rare pediatric developmental disorders in the United Kingdom and Ireland subsequently demonstrated the diagnostic value of combining exome sequencing with detailed clinical assessment and iterative variant interpretation [2]. Diagnostic outcomes nevertheless depend on referral indications, family structure, available phenotypic information, and the scope of analysis.

Inherited metabolic disorders (also termed inborn errors of metabolism [IEM]) occupy a unique place within this landscape. The 2021 international classification includes more than 1400 distinct conditions, most of which affect multiple organ systems [3]. Two features make an accurate molecular diagnosis especially important. First, IEMs often present with overlapping, nonspecific features, such as developmental delay, hypotonia, organomegaly, and dysmorphism, making phenotype-driven targeted testing challenging. Second, unlike many other pediatric rare diseases, a substantial proportion of IEMs are treatable, allowing genomic confirmation to directly guide enzyme replacement therapy, dietary modification, cofactor supplementation, or transplantation [4,5]. WES, particularly alongside detailed biochemical phenotyping, has shortened the diagnostic odyssey for many of these children [4].

Genome-wide testing may also identify two or more independent molecular diagnoses in a single patient. Posey and colleagues reported that 101 of 2076 molecularly diagnosed patients (4.9%) carried variants in two or more disease-associated loci, with the distinct diagnoses often affecting different organ systems [6]. The prevalence is not fixed: it was substantially higher in a consanguineous neurodevelopmental cohort [7], reflecting the influence of ascertainment and population structure. Clinically, a single diagnosis that fails to explain the full phenotype may warrant evaluation for an additional disorder.

The frequency of multiple molecular diagnoses specifically among pediatric-onset IEMs remains poorly defined. Available estimates largely come from mixed-indication referral populations. In Taiwan, clinical WES is often outsourced, and the information available for subsequent review depends on the reports and data released by the testing providers. We reviewed eight years of outsourced genomic testing at a single pediatric genetics center and selected 13 patients with reported or suspected IEMs, including lysosomal storage diseases. We described their molecular findings, examined how the proportion of multi-locus findings changed under different diagnostic definitions, and summarized the pre-sequencing workup and clinical management.

## 2. Materials and Methods

### 2.1. Study Design and Setting

We performed a retrospective observational study at the Division of Pediatric Genetics and the International Rare Disease Centre of MacKay Memorial Hospital, Taipei, a tertiary referral center for inherited metabolic diseases in northern Taiwan. The hospital had no in-house exome facility during the study period, so clinical genomic testing was outsourced. Between 2018 and 2019, sequencing was performed through a government-funded research program, with family structure described in later case summaries as noted in Section 2.3. Thereafter (2020–2025), testing was arranged through commercial providers; funding was not uniformly self-pay. P2-05 was recorded as supported by research funds without a patient charge. Reporting followed the STROBE recommendations for observational research [8]. The study protocol was approved by the Institutional Review Board of MacKay Memorial Hospital (21MMHIS109e) and was conducted in accordance with the Declaration of Helsinki.

### 2.2. Case Ascertainment and Eligibility

Cases were identified from the divisional registry of outsourced next-generation sequencing (NGS) and related genetic tests. We reviewed 13 selected patients referred between January 2018 and December 2025 with a reported or suspected metabolic diagnosis, including lysosomal storage diseases, mitochondrial disorders, and congenital disorders of glycosylation, using the International Classification of Inherited Metabolic Disorders as a framework [3]. All 13 were linked to their original registry entries. The registry contains different testing modalities, undated entries, and a duplicated referral; the parent-cohort counts previously stated as 227 referrals and 91 diagnoses could not be reconciled to this workbook without the original inclusion and diagnostic adjudication list and are not used as denominators here. This exploratory selection retained P2-10, whose metabolic finding was a *COG4* variant of uncertain significance (VUS), and P2-12, whose findings involved candidate mitochondrial solute-carrier genes; their inclusion does not establish a confirmed IEM. Their diagnostic uncertainty is addressed separately in Section 2.6 and the Limitations subsection. These data do not estimate the diagnostic yield of sequencing for suspected IEMs.

At our center, WES was initially used mainly when conventional biochemical, enzymatic, cytogenetic, or targeted molecular investigations were inconclusive, or when an established diagnosis did not explain the full phenotype. Later in the study period, WES was used more broadly as a molecular testing strategy, including for patients with a biochemical or newborn-screening indication. Thus, a metabolic diagnosis could be suspected or established before WES, with sequencing used for molecular characterization or assessment of additional features. Biochemical confirmation and clinically indicated treatment were not contingent on receiving the WES result. The transition was gradual rather than defined by a prespecified calendar cutoff. The original registry records WGS for P2-06 and P2-07, so this selected series is not presented as a uniform WES cohort.

The primary diagnosis refers to the diagnosis or finding most closely related to the pre-sequencing referral indication. An additional diagnosis refers to a separate, phenotype-relevant disorder, whereas an additional finding denotes an unresolved variant or candidate association. These terms describe the clinical relationship to the referral and do not refer to American College of Medical Genetics and Genomics (ACMG) secondary findings, which arise from deliberate analysis of medically actionable genes unrelated to the testing indication. Uncertain additional findings are labeled explicitly.

Pediatric onset was defined as documented onset of disease-related clinical or biochemical manifestations before 18 yr of age, regardless of age at genomic testing. Accordingly, one patient who underwent WES at 23.1 yr of age was included because generalized seizures and psychomotor regression had begun in early childhood.

### 2.3. Outsourced Genomic Testing

Five patients were referred through the National Health Research Institutes (NHRI) research program and eight through commercial pathways. Later case summaries describe four research-program cases as trios and one as a singleton, and all commercial cases as singletons; family structure was not independently established for every case. The registry identifies P2-06 and P2-07 as WGS, whereas later summaries use WES. Table S3 lists providers by test type and retains the earlier codes for cross-reference. P2-03 has Compass Bioinformatics in the WES field and Ao-Ji in the Sanger field; P2-02 and P2-13 have Compass Bioinformatics in the WES field, and Jing-Ji appears in the aCGH field for P2-13. These records may identify a referral intermediary rather than the laboratory that generated or analyzed the sequence data. Ao-Ji and Jing-Ji are romanized provider labels from the registry.

This retrospective review used the referral registry and clinical case summaries. Historical capture chemistry, sequencing platforms, depth, coverage, reference builds, and analytical pipelines were not recorded consistently, so laboratory-specific analytical performance was not compared. Primary sequence data were not reprocessed as part of this study.

When only a single pathogenic allele was identified in an autosomal recessive gene despite a concordant enzymatic phenotype, complementary whole-genome sequencing was requested. In one patient (P2-05), this identified a deep intronic *GALNS* variant that had not been captured by WES. Parental Sanger sequencing was performed whenever samples were available to determine inheritance and variant phase. In several families, this was not feasible because of parental death or loss to follow-up; in these cases, inheritance was recorded as presumed.

### 2.4. Clinical Data Extraction

Two investigators independently extracted data from electronic and paper records using a structured form. Variables included demographics and ancestry; age at first documented symptom and at genomic-test referral; presenting phenotype by organ system; pre-sequencing investigations, recorded narratively because documentation was too heterogeneous to tabulate (basic biochemistry, tandem mass spectrometry newborn screening, enzyme assays, urinary glycosaminoglycan analysis, brain imaging, echocardiography, karyotyping, chromosomal microarray, targeted single-gene or panel testing, and electroencephalography); biochemical and enzymatic findings with laboratory-specific reference intervals; treatment and its timing; and clinical status at the most recent follow-up.

An investigation was considered performed only when a dated result was available in the medical record and was further categorized as contributing to the final diagnosis, abnormal but nonspecific, or normal. Discrepancies between investigators were resolved by joint review of the source records with a senior clinical geneticist. The complete abstracted clinical information for each patient, including presentation, pre-exome investigations, molecular findings, treatment, and outcome, is provided in Table S1.

### 2.5. Review of Variant Descriptions and Recorded Classifications

Variant descriptions and classifications were reviewed using the available reports and case-level summaries. Table S2 provides the recorded RefSeq accession and version, coding-DNA description, protein consequence, zygosity, and evidence notes. Human Genome Variation Society (HGVS) conventions were applied where the source information was sufficient [9]. Predicted protein consequences are enclosed in parentheses, and an undetermined consequence is shown as p.?. Incomplete or internally inconsistent descriptions are explicitly identified rather than reconstructed from a protein change alone. The recorded classifications are presented with reference to the ACMG/Association for Molecular Pathology (AMP) framework [10]. Population databases, ClinVar, prediction tools, and published functional evidence can inform interpretation [11,12,13,14,15,16], but the available documentation does not permit a new, fully reproducible classification of every variant.

A biochemical diagnosis and a variant classification were evaluated separately. A characteristic enzyme defect may support the clinical diagnosis, but it does not by itself establish the pathogenicity of a particular VUS or identify an undetected second allele. Reported P/LP labels were retained as source classifications unless a specific correction was supported. Unresolved classifications and gene–disease associations were excluded from the strict multi-locus count. Table S2 distinguishes recorded evidence from unresolved annotations. When classifications differ between the original registry and later case summaries, both are identified; the later labels are not treated as independently validated reclassifications.

### 2.6. Definitions of Reported Findings and Multi-Locus Diagnoses

A reported gene-level finding denotes a gene and its variant or variants recorded as relevant to the clinical assessment. It is not necessarily a confirmed diagnosis. A molecular diagnosis requires an established gene–disease relationship and P/LP variants in the expected zygosity and inheritance pattern. A clinically or biochemically supported metabolic diagnosis with incomplete molecular evidence is described separately. In particular, the single *HGSNAT* variant in P2-06 does not establish a biallelic molecular diagnosis.

The inclusive multi-locus analysis counted patients with findings at two distinct loci, including additional VUSs and candidate gene–disease associations. Sensitivity analyses first excluded the candidate-gene findings in P2-12 and then required P/LP variants in the expected zygosity at both loci. The 13 selected patients remained the denominator in each analysis so that the effect of the counting rule could be examined. Unlike the strict analysis, the inclusive definition is broader than the molecular-diagnosis definition used by Posey et al. [6]. Findings were counted at the gene level; two variants in one gene contributed two variants but only one gene-level finding.

Cytogenetic and copy number findings identified before WES were described separately and were not included in the sequence-based multi-locus count. These comprised a maternally inherited 7q duplication, a 4q23–q24 deletion, and a complex chromosomal rearrangement. This operational exclusion does not imply that the findings were clinically unimportant. The *ZNF469* findings in P2-09, comprising one pathogenic and one likely benign variant, were reported as carrier findings and were excluded from the 18 gene-level findings and 27-variant summary. No biallelic *ZNF469* diagnosis was established.

### 2.7. Reporting Intervals and Clinical Management

The reporting interval was calculated as the calendar-day difference between the registry submission and report dates. Four commercial episodes had both dates: P2-05, 117 days; P2-06, 28 days; P2-07, 43 days; and P2-08, 27 days. P2-06 and P2-07 are recorded as WGS. P2-05 underwent WES and complementary WGS in the clinical summary, but the registry does not identify which report defines the end of its 117-day interval. These intervals are therefore summarized as mixed genomic-testing episodes, not as a WES-only turnaround time. Symptom-onset and treatment-start dates remained too incomplete for analysis of diagnostic delay or time from sequencing to treatment.

Clinical management was summarized descriptively from the records, including therapy, genetic counseling, parental testing, prenatal diagnosis, rare disease registration or disability certification, and multidisciplinary care. Receipt of treatment was distinguished from treatment initiated because of sequencing. Where treatment preceded sequencing or the temporal relationship was unclear, it was not attributed to the genomic result.

### 2.8. Statistical Analysis

Statistical analysis was descriptive. Continuous variables are presented as median, interquartile range, and range; categorical variables are presented as counts and percentages with the denominator specified. Multi-locus proportions are accompanied by exact Clopper–Pearson 95% confidence intervals. The published 4.9% estimate is provided for context only [6]. No fold comparison or formal hypothesis test was used because ascertainment and diagnostic definitions differed. The unpublished parent-cohort percentage was omitted because its numerator, denominator, and counting criteria could not be verified from the available case-level dataset.

Missing data were not imputed. Variables with incomplete documentation are reported using the available denominator and identified accordingly. Statistical analyses were performed using MedCalc Statistical Software version 23.6.3 (MedCalc Software Ltd., Ostend, Belgium; https://www.medcalc.org (accessed on 12 October 2026); 2026). Percentiles were calculated using the program’s default rank-based method, in which the percentile at cumulative proportion *p* corresponds to the observation of rank *p* × *n* + 0.5, with linear interpolation between adjacent observations [17]. Because different percentile definitions can produce appreciably different quartiles in small samples, the reported interquartile ranges should be interpreted using this definition.

### 2.9. De-Identification

Patients are identified by study codes P2-01 to P2-13. The linkage to personal identifiers is retained only in the institutional registry. Treating clinicians and external care institutions are not named, and dates are generally reported at quarter-year resolution. Provider names in Table S3 describe testing pathways and are not patient identifiers. No patient images are included.

### 2.10. Use of Generative Artificial Intelligence

Generative AI tools, including Claude (Opus 5.5; Anthropic) and ChatGPT (Astra 6.0; OpenAI), were used to assist with language editing, organization of the revision, literature searches, reference formatting, and checks for numerical consistency. The authors are responsible for reviewing the proposed changes against source reports and records and for the final scientific content and interpretation.

## 3. Results

### 3.1. Cohort Characteristics

**Figure 1:**
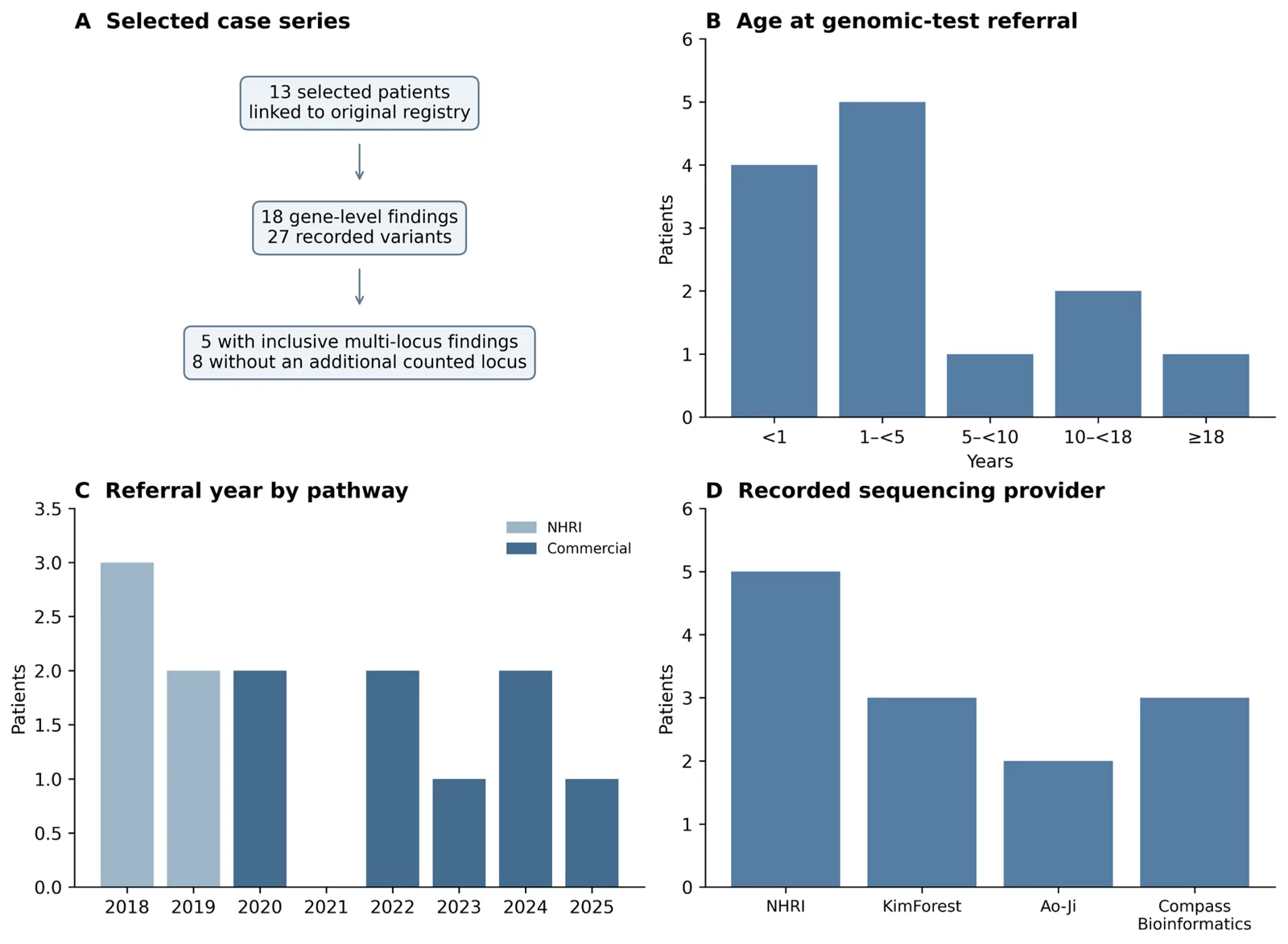
Selected cases and referral characteristics. (**A**) Thirteen registry-linked patients with reported or suspected metabolic diagnoses. Five had inclusive multi-locus findings and eight had no additional counted loci. Parent-cohort diagnostic-yield counts remain unreconciled and are not plotted. (**B**) Age at genomic-test referral. (**C**) Referral year by pathway, not patient payment status. (**D**) Providers recorded in the relevant sequencing fields. The provider may be an intermediary; Table S3 distinguishes WES, WGS, Sanger, and aCGH entries. WES, whole-exome sequencing; WGS, whole-genome sequencing; aCGH, array comparative genomic hybridization.

The series comprised 13 selected patients linked to the original referral registry (Figure 1A; Table S1). Ten were male and three were female. Median age at genomic-test referral was 2.7 yr (IQR, 0.7–9.6; range, 0.0–23.1), recalculated from the recorded birth and submission dates. Four patients were referred before age 1 yr and five at age 1 to <5 yr (Figure 1B; Table 1). Five were referred through the NHRI program in 2018–2019 and eight through commercial pathways in 2020–2025 (Figure 1C,D). P2-05 had research funding despite a commercial pathway. No patient in this subset was referred in 2021. Family-structure counts derive from later summaries and retain the unresolved P2-11 designation described in Section 2.3. Twelve families were of Han Chinese Taiwanese ancestry and one of mixed Taiwanese and European ancestry.

Mucopolysaccharidoses were the largest disease group (4/13), followed by the recorded mitochondrial group (2/13). The latter includes the candidate findings in P2-12. Each remaining disease group comprised one patient (Figure S1); the glycosylation category reflects the uncertain *COG4* finding in P2-10. P2-05 and P2-07 entered metabolic follow-up after abnormal newborn screening. Other patients were assessed for symptomatic disease, multisystem abnormalities, or an incompletely explained phenotype.

### 3.2. Molecular Diagnoses and Variant Spectrum

The 13 patients had 18 reported gene-level findings comprising 27 variants (Table 2; Table S2). The recorded inheritance categories were autosomal recessive for nine findings, autosomal dominant for four, and X-linked for five. These categories describe the source annotations and do not establish the validity of candidate gene–disease associations. The *PTPN11* variant in P2-03 was presumed, rather than confirmed, de novo. The *ZSWIM6* finding in P2-10 was recorded as de novo; its incomplete annotation is identified in Table S2. Original-registry classifications differ from later summaries for G6PD in P2-01, both *SLC6A9* variants in P2-09, *SLC6A8* in P2-11, and both *SLC25A41* variants in P2-12. Table S2 records these conflicts without assuming that a reclassification was completed.

**Figure 2:**
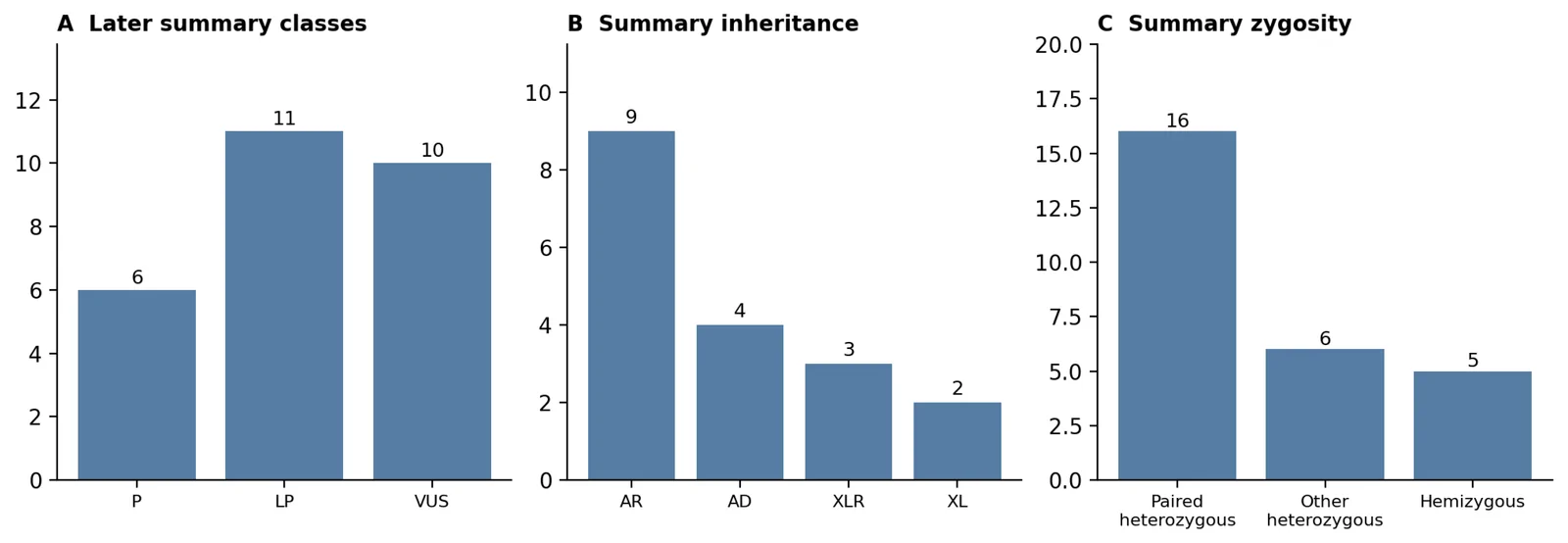
Recorded variant classifications and inheritance annotations. (**A**) Later case-summary labels for 27 variants; six differ from the original registry and require adjudication. (**B**) Inheritance annotations for 18 gene-level findings, including uncertain and candidate findings. (**C**) Recorded zygosity groups. Paired heterozygous findings are not assumed to be proven in trans in every case. No homozygous variant was reported. P, pathogenic; LP, likely pathogenic; VUS, variant of uncertain significance; AD, autosomal dominant; AR, autosomal recessive; XL, X-linked; XLR, X-linked recessive.

The later case summaries assigned pathogenic labels to six variants (22.2%), likely pathogenic labels to 11 (40.7%), and uncertain labels to 10 (37.0%) (Figure 2A). These counts describe the later summary labels and do not resolve the six variant-level discrepancies with the original registry or establish 17 validated causal variants. Sixteen variants were recorded in paired heterozygous findings in recessive genes, six as other heterozygous findings, and five as hemizygous findings. Phase was not independently established for every pair. No homozygous variant was reported (Figure 2C). The *GALNS* c.423-862C>T finding was identified by complementary genome sequencing after WES reported one *GALNS* variant.

P2-09 had one *ZNF469* variant recorded as pathogenic, p.(Arg3090Ter), and another recorded as likely benign, p.(Pro1363Arg). No corneal thinning, blue sclerae, or keratoconus was documented. These findings were recorded as carrier findings rather than a biallelic diagnosis and were excluded from the multi-locus count.

P2-11 had a heterozygous *PIEZO2* p.(Val1660Ala) VUS. Mild joint contractures, intellectual disability, and corrected esotropia were documented, but the variant remained uncertain. The available description lacked a coding DNA change, and segregation and functional studies were not available.

### 3.3. Multi-Locus Findings Under Different Definitions

**Figure 3:**
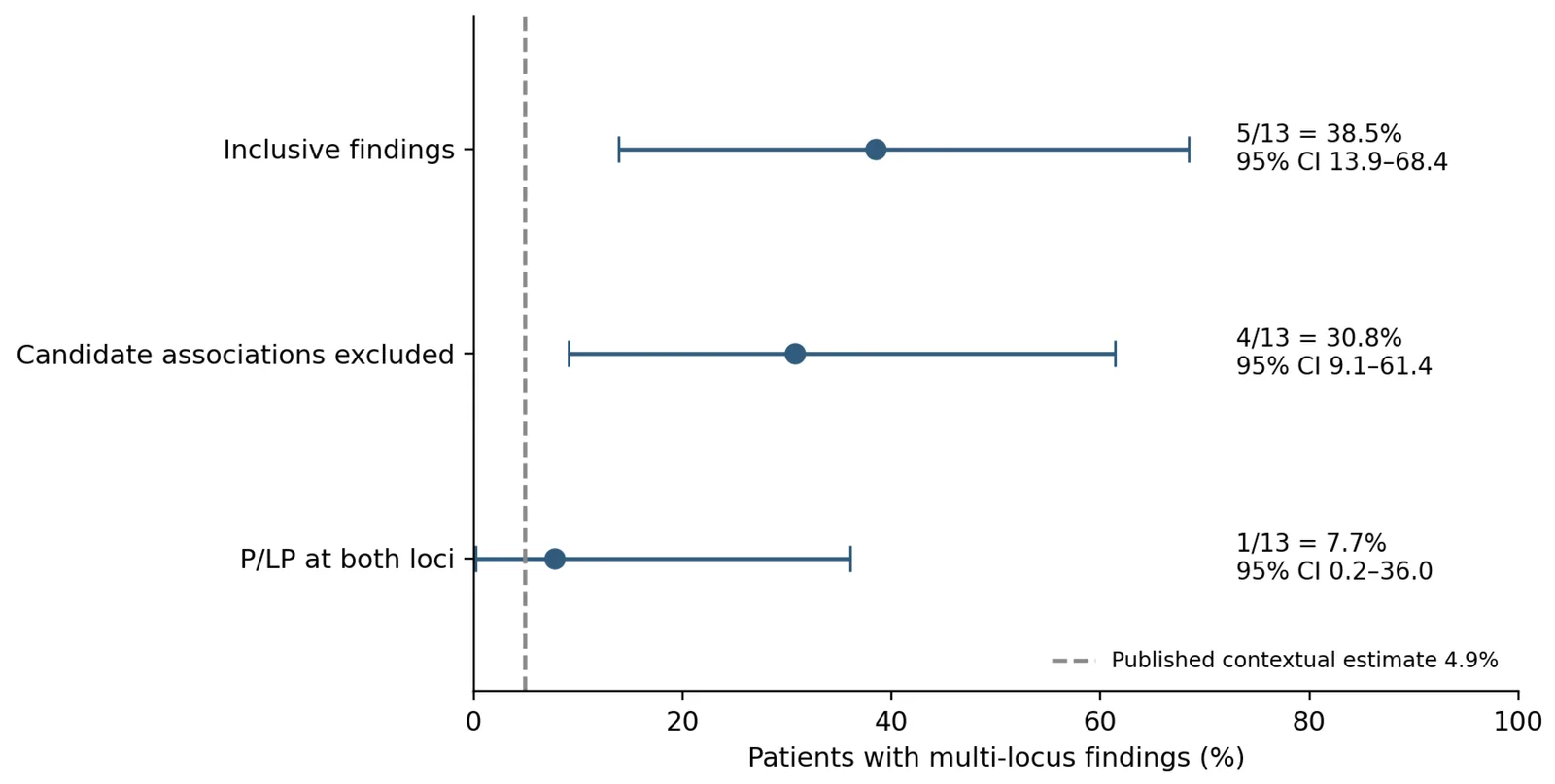
Effect of the multi-locus counting rule. The point estimates and exact 95% confidence intervals are shown for the inclusive definition (5/13), exclusion of candidate gene–disease associations (4/13), and the strict P/LP definition (1/13). The dashed line marks the published 4.9% estimate among 2076 molecularly diagnosed patients in the Posey et al. series [6]. It is a contextual benchmark, not a matched control. P/LP, pathogenic or likely pathogenic.

Five of 13 patients met the inclusive multi-locus definition (38.5%; exact 95% CI, 13.9–68.4%; Table 3). Excluding P2-12, whose findings involved candidate gene–disease associations, left four of 13 (30.8%; 95% CI, 9.1–61.4%). Requiring P/LP variants in the expected zygosity at both loci left P2-03 alone (1/13; 7.7%; 95% CI, 0.2–36.0%; Figure 3). These estimates use the same exploratory denominator and should not be read as three estimates of confirmed disease prevalence.

The counted pairs were *MMUT* and *PTPN11* in P2-03, *ABCG5* and *FLNA* in P2-08, *ZSWIM6* and *COG4* in P2-10, *SLC6A8* and *PIEZO2* in P2-11, and *SLC25A41* and *SLC25A5* in P2-12. P2-03 presented with neonatal metabolic acidosis, hyperammonemia, methylmalonic aciduria, and an atrial septal defect. P2-08 had xanthomas, hypercholesterolemia, and pulmonary atresia with an intact ventricular septum. P2-10 had a neurodevelopmental presentation with an additional *COG4* VUS. No causal contribution of the uncertain additional findings was established by this analysis.

Three patients had cytogenetic findings before WES: a complex rearrangement in P2-11, a maternally inherited 7q duplication in P2-09, and a 5.1 Mb 4q23–q24 deletion in P2-13. These were described alongside the sequence findings but were excluded from the sequence-based multi-locus count according to Section 2.6.

### 3.4. Pre-Sequencing Investigation and Age at Referral

Pre-sequencing investigations included biochemical tests, enzyme assays, urinary glycosaminoglycan analysis, imaging, and selected cytogenetic or targeted molecular tests (Table S1). The records describe *PHOX2B* testing in P2-01, *IDS* testing in P2-07, and spinal muscular atrophy screening in P2-09 and P2-10. None of these tests established the subsequently reported diagnosis. The registry records *IDS* c.1181-15C>A after an uninformative targeted test and lists the genomic test as WGS; it is described here as an intronic variant, without implying that it lies deep within the intron. Documentation was insufficient for a complete patient-by-test matrix.

Referral age ranged from the neonatal period to 23.1 yr (Figure S2). WES was requested on day 2 of life in P2-03 during evaluation of an acute metabolic presentation. P2-11 and P2-12 were referred at 14.8 and 23.1 yr, respectively. Recalculation of the four registry date pairs gave reporting intervals of 27, 28, 43, and 117 days (median, 35.5 days; IQR, 27.5–80.0; Table 4). These are mixed genomic-testing episodes.

### 3.5. Clinical Management

Table 4 summarizes documented management. Enzyme replacement therapy was recorded for P2-02, P2-05, and P2-07; P2-07 also underwent hematopoietic stem cell transplantation. In P2-05, therapy began in 2021, before WES in 2023. Treatment receipt therefore cannot be interpreted as a treatment change caused by WES. The records did not support a reconciled count of patients who started disease-specific therapy because of the WES result.

**Figure 4:**
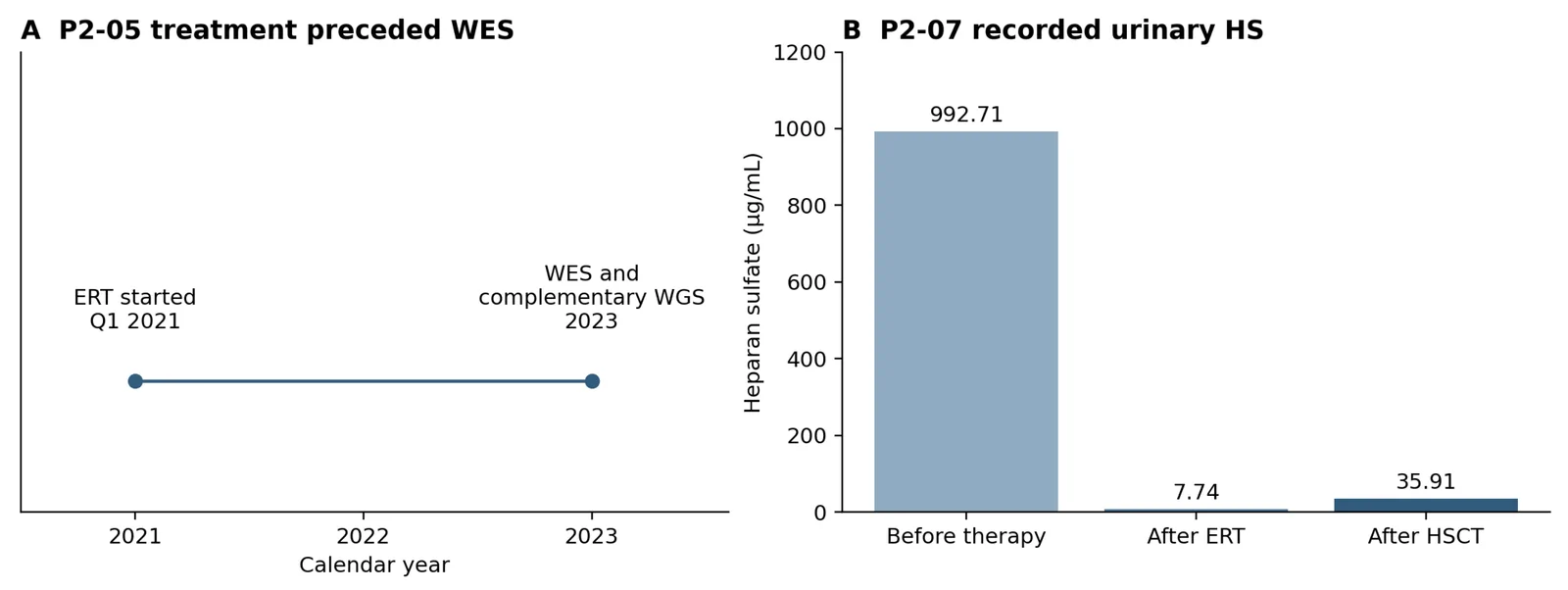
Treatment chronology and selected biomarker observations. (**A**) P2-05 started elosulfase alfa in Q1 2021 and underwent WES with complementary genome sequencing in 2023, demonstrating that treatment preceded sequencing. (**B**) Recorded urinary heparan sulfate measurements for P2-07 before therapy, after ERT, and after HSCT were 992.71, 7.74, and 35.91 µg/mL, respectively. These are individual observations, without an untreated comparator, and do not isolate the effects of ERT and HSCT. The plotted values follow the clinical case summary. ERT, enzyme replacement therapy; HSCT, hematopoietic stem cell transplantation.

In P2-07, the recorded urinary heparan sulfate concentration decreased after ERT and remained below the pretreatment value after transplantation; donor chimerism was documented by short tandem repeat analysis. P2-05 continued ERT that had begun before WES (Figure 4). P2-06 received supportive care, with the completeness of the molecular and biochemical confirmation remaining a limitation.

## 4. Discussion

The inclusive multi-locus proportion was 38.5% (5/13; 95% CI, 13.9–68.4%), whereas the strict P/LP-based proportion was 7.7% (1/13; 95% CI, 0.2–36.0%). Posey et al. reported 4.9% among molecularly diagnosed patients in a larger clinical exome series [6]. These values describe differently selected populations under different diagnostic rules. The present data do not establish a higher prevalence of confirmed multi-locus disease in pediatric IEMs.

Diagnostic certainty accounts for much of the difference between the inclusive and strict estimates. The *FLNA*, *COG4*, and *PIEZO2* findings remain VUSs, and the *SLC25A41* and *SLC25A5* findings were reported in the context of candidate gene–disease associations [18,19]. Keeping the denominator fixed at 13 makes the effect of the counting rule visible, but it does not resolve uncertainty about eligibility: P2-10 and P2-12 would require reassessment in a cohort restricted to established metabolic diagnoses. The inclusive count should therefore be regarded as a description of findings warranting review, not as five confirmed dual diagnoses.

Selection for WES changed over the study period. Earlier referrals commonly followed inconclusive conventional testing or a mismatch between an existing diagnosis and the remaining phenotype; broader use later included molecular characterization of biochemically supported disorders. Together with the restriction to registry cases with reported diagnoses, this limits extrapolation to all children referred for possible IEMs. Cytogenetic findings were excluded operationally from the sequence-based count, although they may still contribute to individual phenotypes. No homozygous variant was reported. The absence of reported consanguinity does not exclude homozygosity, including that associated with founder variants or population-specific allele frequencies, and this small cohort cannot establish a population-genetic explanation for the observed pattern.

When an established diagnosis does not explain the full phenotype, the remaining features should prompt consideration of an additional diagnosis. Other explanations include variable expressivity, complications of the known disorder, acquired disease, environmental effects, or an unrelated finding. In P2-03, the pathogenic *PTPN11* finding supported a coexisting Noonan syndrome diagnosis and made the cardiac finding relevant to further surveillance. By contrast, the additional *FLNA*, *COG4*, and *PIEZO2* VUSs identify questions for follow-up rather than established causes of the residual features. A heterozygous *COG4* VUS should not be equated with Saul–Wilson syndrome solely because that gene is associated with the disorder; the original series involved the recurrent p.Gly516Arg substitution [20].

Outsourced testing limited the analysis because primary sequence files were not available to the study team, preventing independent reprocessing. Published reanalysis studies show that revisiting unsolved exomes can yield additional diagnoses [21,22]. In P2-05, complementary genome sequencing identified a second *GALNS* finding outside the initial exome result. Genome sequencing can identify diagnoses that require intronic or structural information beyond standard exome analysis [23]. When biochemical findings and WES results are discordant, further testing should be selected according to the suspected missed variant type and the data available. Provider agreements that specify data access and reanalysis arrangements may facilitate this work.

Clinical usefulness also needs to be separated from treatment attribution. Genomic findings may inform counseling, surveillance, or further testing, but treatment may already have been started on biochemical grounds, as in P2-05. Biomarker observations after treatment in P2-07 document the clinical course but cannot isolate the contribution of sequencing, ERT, or transplantation. A retrospective management analysis requires dated treatment decisions and an explicit link to the molecular result before a change can be attributed to sequencing.

### Limitations

This single-center retrospective series is small and highly selected. The analysis conditions on registry cases with reported diagnoses and further includes uncertain metabolic findings; it does not estimate the prevalence of multi-locus disease in an unselected population. The complete parent-cohort denominator and diagnostic-status assignments remain unadjudicated. The source registry records WGS for two included patients; therefore, these findings cannot be interpreted as WES-specific outcomes. Referral practices, report content, and completeness of clinical documentation varied across the eight-year period.

Diagnostic certainty varied across cases. The *FLNA*, *COG4*, and *PIEZO2* VUSs and the candidate-gene findings in P2-12 do not establish additional diagnoses; only one *HGSNAT* allele was recorded in P2-06. Incomplete variant annotations and six classification differences between the registry and later summaries are detailed in Table S2. These issues were reflected in the sensitivity analyses. Family-structure and treatment-timing information was also incomplete, and reporting intervals combined different genomic-testing episodes. Published benchmark cohorts differ in selection and diagnostic definitions, limiting direct comparison.

## 5. Conclusions

In this selected metabolic-related cohort, additional findings were common under an inclusive definition, but only one of 13 patients met the strict P/LP-based multi-locus criterion using the recorded classifications. This proportion is imprecise and does not establish an excess over published clinical exome series. Unexplained features should prompt clinical reassessment and consideration of an additional diagnosis, with careful separation of confirmed disease, VUSs, and candidate associations. Access to primary data and appropriate complementary testing can support further evaluation.

## Figures and Tables

**Table 1:** Baseline characteristics of the 13 patients in the metabolic-disorder subset (outsourced genomic testing, 2018–2025).

Characteristic	Value
Total patients, *n*	13
Reported gene-level findings, *n*	18
Variants in counted findings, *n*	27
***Sex***	
Male, *n* (%)	10 (76.9)
Female, *n* (%)	3 (23.1)
***Age at genomic-test referral***	
Median (IQR), yr	2.7 (0.7–9.6)
Range, yr	0.0–23.1
***Age group at genomic-test referral, n (%)***	
<1 yr (infant)	4 (30.8)
1–<5 yr (toddler/preschool)	5 (38.5)
5–<10 yr (school age)	1 (7.7)
10–<18 yr (adolescent)	2 (15.4)
≥18 yr (adult age, pediatric-onset)	1 (7.7)
***Referral period, n (%)***	
2018–2019 (research-funded)	5 (38.5)
2020–2025 (commercial pathways)	8 (61.5)
***Recorded sequencing provider, n (%)***	
Research-funded program	5 (38.5)
KimForest	3 (23.1)
Ao-Ji	2 (15.4)
Compass Bioinformatics	3 (23.1)
Family structure in later summaries *	
Singleton	9 (69.2)
Trio	4 (30.8)
***Disease group, n (%)***	
Mucopolysaccharidosis (MPS)	4 (30.8)
Mitochondrial disorders	2 (15.4)
Organic acidemia	1 (7.7)
Glycogen storage disease	1 (7.7)
Amino-acid metabolism	1 (7.7)
Creatine metabolism	1 (7.7)
Lipid storage (sitosterolemia)	1 (7.7)
Suspected glycosylation disorder	1 (7.7)
Other IEM (G6PD deficiency)	1 (7.7)
***Inclusive multi-locus findings***	
No additional counted locus, *n* (%)	8 (61.5)
Additional counted locus, *n* (%)	5 (38.5)
***Family ancestry***	
Han Chinese (Taiwan)	12 (92.3)
Mixed Taiwanese/European	1 (7.7)

Note. IQR, interquartile range; MPS, mucopolysaccharidosis. Counts refer to 13 unique patients unless stated otherwise. The 18 reported gene-level findings comprise 27 variants, including one *GALNS* variant detected only by complementary whole-genome sequencing. Multi-locus findings use the inclusive definition in Section 2.6. Disease groups include suspected categories for P2-10 and P2-12. * Later case summaries record nine singleton and four trio cases; the original registry does not establish family structure. P2-06 and P2-07 are listed as whole-genome sequencing. The P2-11 family-structure designation is uncertain. IEM, inherited metabolic disorder; G6PD, glucose-6-phosphate dehydrogenase. Ao-Ji is a romanized registry provider label. Shading aids reading; bold and italic group labels identify sections of the table.

**Table 2:** Reported gene-level findings in 13 patients.

Patient	Disease or Reported Finding	Gene	Recorded Zygosity	Inh.	Class *	Inclusive Pair
P2-01	G6PD deficiency	*G6PD*	Het	XLR	LP	No
P2-02	MPS VII (Sly)	*GUSB*	Compound het	AR	P/VUS	No
P2-03	MMA mut(0)	*MMUT*	Compound het	AR	LP/LP	Yes
P2-03	Noonan syndrome	*PTPN11*	Het	AD	P	Additional
P2-04	GSD Ib	*SLC37A4*	Compound het	AR	VUS/LP	No
P2-05	MPS IVA (Morquio A)	*GALNS*	Compound het	AR	P/VUS	No
P2-06	Suspected MPS IIIC; one allele	*HGSNAT*	Het ‡	AR	P	No
P2-07	MPS II (Hunter, severe)	*IDS*	Hemi	XLR	VUS	No
P2-08	Sitosterolemia 2	*ABCG5*	Compound het	AR	P/VUS	Yes
P2-08	Additional *FLNA* VUS	*FLNA*	Hemi	XL	VUS	Additional
P2-09	Glycine encephalopathy	*SLC6A9*	Compound het	AR	LP	No
P2-10	*COG4* finding; disease assignment uncertain	*COG4*	Het	AD	VUS	Yes
P2-10	Reported *ZSWIM6* NDD	*ZSWIM6*	Het	AD	P	Additional
P2-11	Cerebral creatine deficiency 1	*SLC6A8*	Hemi	XLR	LP	Yes
P2-11	Additional *PIEZO2* VUS	*PIEZO2*	Het	AD	VUS	Additional
P2-12	Candidate mitochondrial finding	*SLC25A41*	Compound het	AR	LP	Yes
P2-12	Candidate mitochondrial finding	*SLC25A5*	Hemi	XL	VUS	Additional
P2-13	Mitochondrial complex I deficiency	*NDUFA10*	Compound het	AR	LP/LP	No

Note. AD, autosomal dominant; AR, autosomal recessive; LP, likely pathogenic; P, pathogenic; VUS, variant of uncertain significance; XL, X-linked; XLR, X-linked recessive. * The class column preserves later case-summary labels. Conflicting original-registry labels are listed in Table S2; no new reclassification is implied. Full transcript-based descriptions and unresolved annotations are listed in Table S2. Additional *FLNA*, *COG4*, and *PIEZO2* findings remain uncertain. *SLC25A41* and *SLC25A5* findings are candidate associations. The two *SLC25A5* variants were reported as hemizygous in one male and do not constitute compound heterozygosity. *ZNF469* carrier findings in P2-09 are not counted. Inh., inheritance; Het, heterozygous; compound het, compound heterozygous; Hemi, hemizygous; MPS, mucopolysaccharidosis; MMA, methylmalonic aciduria; GSD, glycogen storage disease; NDD, neurodevelopmental disorder; G6PD, glucose-6-phosphate dehydrogenase. ‡ Only one *HGSNAT* allele was identified; a biallelic molecular diagnosis was not established. Italicized symbols denote human genes. Row shading is used only to aid reading.

**Table 3:** Five patients with additional findings under the inclusive definition.

Patient	Primary Finding	Additional Finding	Later Summary Classes	Interpretation
P2-03	*MMUT*-related methylmalonic aciduria	*PTPN11*-related Noonan syndrome	LP/LP; P	Meets strict definition using source classes; *PTPN11* de novo status unconfirmed.
P2-08	*ABCG5*-related sitosterolemia	*FLNA* VUS	P/VUS; VUS	Cardiac contribution uncertain; segregation and functional evidence unavailable.
P2-10	Reported *ZSWIM6* neurodevelopmental finding	*COG4* VUS	P; VUS	*COG4* does not establish Saul–Wilson syndrome; metabolic eligibility and *ZSWIM6* annotation require review.
P2-11	Reported *SLC6A8* creatine deficiency	*PIEZO2* VUS	LP; VUS	*SLC6A8* class conflicts: LP in later summary; benign in original registry. *PIEZO2* remains VUS.
P2-12	*SLC25A41* candidate finding	*SLC25A5* candidate finding	LP; VUS	Candidate associations; *SLC25A41* LP/LP in later summary versus likely benign/VUS in original registry.

Note. The additional findings in P2-08, P2-10, and P2-11 are VUSs; P2-12 has candidate gene–disease associations. Only P2-03 met the strict P/LP definition using the recorded classifications. Primary and additional refer to their relationship to the referral phenotype, not to relative pathogenicity or ACMG secondary findings. P, pathogenic; LP, likely pathogenic; VUS, variant of uncertain significance; ACMG, American College of Medical Genetics and Genomics. Italicized symbols denote human genes. Row shading is used only to aid reading.

**Table 4:** Available reporting intervals and documented clinical management.

**Reporting interval for four commercial diagnostic episodes.**	
**Measure**	**Available summary**
Diagnostic episodes with dates	4
Median (IQR), days	35.5 (27.5–80.0)
Range, days	27–117
Scope	Mixed genomic tests; WGS recorded for P2-06 and P2-07
Case intervals, days	P2-05: 117; P2-06: 28; P2-07: 43; P2-08: 27
**Documented management**	***n* (%)**
ERT recorded during follow-up	3 (23.1)
HSCT recorded during follow-up	1 (7.7)
Family genetic counseling recorded	13 (100.0)
Parental Sanger testing completed or recommended	11 (84.6)
Prenatal diagnosis offered	2 (15.4)
Rare disease registration or disability certification	9 (69.2)
Multidisciplinary specialist care recorded	13 (100.0)

Note. The reporting-interval section combines registry reporting intervals: P2-06 and P2-07 are listed as WGS; P2-05 had complementary WGS but its final-report type remains unresolved. These are not WES-only turnaround times. The management section describes care recorded during follow-up; categories overlap and are not mutually exclusive. Therapy may have preceded WES. ERT, enzyme replacement therapy; HSCT, hematopoietic stem cell transplantation; IQR, interquartile range. WES, whole-exome sequencing; WGS, whole-genome sequencing. Shading aids reading; bold type identifies the two sections within this single table.

## Data Availability

De-identified case-level summaries are provided in Tables S1 and S2. The referral registry contains patient identifiers and is not included in the submission. This analysis used clinical records and reported findings; primary sequence data were not reprocessed. Further information may be requested from the corresponding authors, subject to institutional review board approval and applicable data-protection requirements.

## Supplementary Materials

The following supporting information can be downloaded at: https://www.mdpi.com/article/10.3390/diagnostics16203356/s1, Table S1, Detailed per-patient clinical summaries (*n* = 13); Table S2, Recorded descriptions of 27 variants across 18 gene-level findings; Table S3, Recorded provider pathways and technical-information availability; Figure S1, Disease groups used for the exploratory analysis; Figure S2, Age at genomic-test referral by patient. Reference [24] is cited in the Supplementary Materials.
