# Supplementary material for: Multi-Locus Molecular Findings in Pediatric-Onset Inherited Metabolic Disorders: An Eight-Year Series of Outsourced Genomic Testing in Taiwan
Source: Diagnostics (Basel). 2026 Oct 15;16(20):3356. doi: 10.3390/diagnostics16203356 (PMC13703565; doi:10.3390/diagnostics16203356)
Supplement: Supplementary file 1 [file diagnostics-16-03356-s001.zip › diagnostics-4587516-supplementary.pdf]

Supplementary Materials

Multi-locus molecular findings in pediatric-onset inherited metabolic disorders: an eight-year series of outsourced genomic testing in Taiwan

Chung-Lin Lee, Ya-Hui Chang, Chih-Kuang Chuang, Huei-Ching Chiu, Yuan-Rong Tu, Yun-Ting Lo, Jun-Yi Wu, Huang-Ying Huang, Hsiang-Yu Lin, Shuan-Pei Lin

Contents

Table S1. Detailed per-patient clinical summaries (*n* = 13)

Table S2. Recorded descriptions of 27 variants across 18 gene-level findings

Figure S1. Disease groups used for the exploratory analysis

Figure S2. Age at genomic-test referral by patient

Table S3. Recorded provider pathways and technical-information availability

Table S1. Detailed per-patient clinical summaries (*n* = 13)

Patient blocks summarize the clinical records using study identifiers only. Provider pathways are detailed separately in Table S3. Reported diagnoses are distinguished from uncertain variants and candidate associations.

Patient P2-01

**Demographics**	Female, born 2018; aged 0.4 years at genomic-test referral; Han Chinese (Taiwan).
**Clinical presentation**	NICU presentation with near-miss sudden infant death syndrome (SIDS); pulmonary hypertension (treated with milrinone and adrenaline); pulmonary hemorrhage; tachycardia (treated with amiodarone); anemia requiring packed red blood cell transfusion; potential sepsis (treated with vancomycin and meropenem); G6PD deficiency identified during routine NICU workup.
**Pre-sequencing workup**	*PHOX2B* gene analysis (2018) for congenital central hypoventilation syndrome: GCN repeats 20/20, no mutation detected. Standard NICU biochemistry, infection workup, and supportive imaging.
**Genomic findings and diagnostic interpretation**	NHRI referral in 2018. The registry submission date is 138 days after the recorded birth date (0.38 yr); the earlier narrative gave day 132. The later summary calls the test singleton WES. *G6PD* p.(Arg489Leu) is labeled pathogenic in the registry and likely pathogenic in the later summary. The original registry incorrectly labels inheritance as autosomal dominant; G6PD is X-linked.
**Treatment and management**	Symptomatic NICU management (cardiopulmonary support, transfusion, empirical antibiotics); no specific IEM-directed therapy required.
**Outcome and follow-up**	Survived NICU admission. Last documented OPD visit at 8 months: alert, feeding well, training standing skill. Long-term follow-up after this visit not documented in available chart.
**Interpretation**	*G6PD* identified by WES performed for NICU multisystem disease (not primarily for metabolic indication); retained in P2 cohort with explicit framing.

Patient P2-02

**Demographics**	Female, born 2015; aged 4.8 years at genomic-test referral; Han Chinese (Taiwan).
**Clinical presentation**	Short stature (consistently <3rd percentile), failure to thrive, hepatosplenomegaly status-post Kasai operation for biliary atresia, dysostosis multiplex, corneal clouding, characteristic facial dysmorphism (flat profile, mid-face hypoplasia, flat nasal bridge, hypertelorism), pigeon chest, joint stiffness, profound intellectual disability, ventilator dependency (BiPAP 10 h/day), atrial septal defect with thick interventricular septum, mitral and aortic regurgitation.
**Pre-sequencing workup**	β-Glucuronidase enzyme assay: 26.67 µmol/g protein/h (reference 255.31–681.67) — markedly reduced. Urinary GAG analysis: DMB ratio 23.06 (reference <20.98); heparan sulfate 0.84 µg/mL (reference <0.78), elevated. Bone survey: vertebral bullet shapes. Cardiac echocardiography. Abdominal imaging post-Kasai. Six-minute walking test 225 m (27.9% of predicted).
**Genomic findings and diagnostic interpretation**	Singleton WES at Compass Bioinformatics (original WES-field provider; former code C) (2020). *GUSB* c.104C>A p.(Ser35Ter) (paternal; pathogenic nonsense) + c.1454C>T p.(Ser485Phe) (maternal; VUS). Compound heterozygous; diagnosis: MPS VII / Sly syndrome.
**Treatment and management**	Enzyme replacement therapy (vestronidase alfa) biweekly. Multidisciplinary care including orthopaedic surgery (right hip Dega osteotomy), pulmonology, ophthalmology.
**Outcome and follow-up**	Alive at most recent follow-up (2026). On ERT >5 years. Severe MPS VII phenotype with progressive cardiac and respiratory involvement; profound ID stable. Corneal clouding progressive.
**Interpretation**	The enzyme deficiency and phenotype support the recorded clinical diagnosis of MPS VII. The missense variant remains VUS; clinical concordance does not independently establish its pathogenicity.

Patient P2-03

**Demographics**	Female, born 2022; WES requested on day 2 of life (age rounded to 0.0 yr); Han Chinese Taiwanese ancestry.
**Clinical presentation**	Term, small-for-gestational-age (BW 2200 g at 41 weeks). Day-2 progressive tachypnoea, severe metabolic acidosis (pH 7.14, HCO_3 4 mmol/L), peak ammonia 472 µg/dL, lactate 152 mg/dL, hypoglycemia, hypocalcemia, hyponatremia, hypotension, coagulopathy, brain edema, seizures, suspected renal tubular acidosis type II, atrial septal defect (0.368 cm).
**Pre-sequencing workup**	Newborn screening (tandem mass spectrometry): C3 18.58, C3/C2 1.09 (both elevated); urine organic acid: methylmalonic acid 2483 µmol/mmol creatinine (markedly elevated, declining to 263 by week 6 with treatment); plasma free carnitine 3.3 (low); total carnitine 28.9 (low). Brain ultrasound: brain edema, decreased RI. Echocardiography. Renal ultrasound. EEG (chaotic regional spikes).
**Genomic findings and diagnostic interpretation**	WES with mitochondrial analysis was requested on day 2 of life in 2022. Compass Bioinformatics is recorded in the WES field and Ao-Ji in the Sanger field. The registry lists *MMUT* c.1106G>A, p.(Arg369His), and c.1677-1G>A. The later clinical summary additionally records *PTPN11* c.794G>A, p.(Arg265Gln), as pathogenic.
**Treatment and management**	Acute: bicarbonate infusion, NPO with parenteral nutrition (no amino acids initially), carglumic acid, intravenous carnitine, vitamin B12, vitamin B complex. Definitive: 14% Anamix infant formula (methionine, threonine, valine free; low isoleucine) initiated day 5; phenobarbital for seizures; oral bicarbonate. 49-day NICU admission.
**Outcome and follow-up**	Discharged for outpatient follow-up. Subsequently transferred to another tertiary center for ongoing care; Noonan-related cardiac and growth follow-up not available at our center. Initial response to metabolic management favorable at discharge.
**Interpretation**	The *MMUT*/*PTPN11* pair meets the strict counting rule using the later summary classes. The *PTPN11* finding is presumed, rather than confirmed, de novo.

Patient P2-04

**Demographics**	Male, born 2022; aged 0.8 years at genomic-test referral; Han Chinese (Taiwan).
**Clinical presentation**	Hypoglycemia (lowest glucose 13 mg/dL), hepatomegaly, lactic acidosis, hypertriglyceridemia (peak 645 mg/dL), elevated transaminases, hyperuricemia, neutropenia (ANC 285/µL during infectious gastroenteritis admission), failure to thrive.
**Pre-sequencing workup**	Plasma amino acids: high α-ketoglutarate, 3-methylglutaric and 3-methylglutaconic acid present. Tandem mass: C0 122.5 (high), C3 10.4 (high), C5OH/C4DC 1.029 (high). Plasma carnitine markedly elevated. Abdominal MRI: hepatomegaly. Echocardiography WNL. Immunoglobulin panel (IgG/A/M/C3/C4): C3 and C4 elevated.
**Genomic findings and diagnostic interpretation**	Singleton WES through Ao-Ji in 2022. The original registry lists *SLC37A4* c.1042_1043delCT, p.Leu348fs*53, and c.898C>T, p.(Arg300Cys). The later summary assigns VUS and LP labels, respectively. The recorded frameshift notation is incomplete because the new amino acid is not specified. The recorded clinical diagnosis is glycogen storage disease type Ib.
**Treatment and management**	Oral Glycosade (uncooked cornstarch derivative): 2 g/kg/day starting dose with nighttime intake 20 g/dose, titrated to blood glucose. Symptomatic management of intercurrent infections.
**Outcome and follow-up**	Alive at most recent follow-up (2026, age 4 years). Doing well, on Glycosade. Growth 25th–50th percentile. Mother had a subsequent pregnancy with chromosomal anomaly leading to therapeutic abortion (2023).
**Interpretation**	Classic GSD Ib presentation with rapid response to dietary management.

Patient P2-05

**Demographics**	Male, born 2020; aged 2.7 years at genomic-test referral; Han Chinese (Taiwan).
**Clinical presentation**	Short stature (<3rd percentile), bilateral simian palms, bilateral inguinal hernia s/p repair at 40 days, dysostosis multiplex (evolving), right genu valgum, mongolian spot on buttock, progressive cardiac involvement (initial MR → dilated left atrium, left and right ventricles → tricuspid and mitral regurgitation), reduced bone mineral density (BMD L1–L4 Z-score –3.6).
**Pre-sequencing workup**	Referred from a regional newborn screening center due to repeated low GALNS enzyme activity. GALNS enzyme assay (leukocyte): 0.0 μmol/g protein/h (reference 5.9–27.8); father 6.1, mother 8.9. Urinary keratan sulfate elevated (10.64 μg/mL, reference <7.9). Serial echocardiography. BMD assessment.
**Genomic findings and diagnostic interpretation**	Testing through Ao-Ji in 2023; the registry records research funding and no patient charge. The submission-to-report interval is 117 days. The registry lists *GALNS* c.953T>G; the later clinical summary identifies p.(Met318Arg) and an additional c.423-862C>T intronic VUS detected by complementary WGS. The registry does not identify whether the recorded endpoint is the WES or WGS report.
**Treatment and management**	Elosulfase alfa began in Q1 2021 at an external center. The recorded infusion schedule and adherence history require confirmation from treatment records. Anti-drug antibody results were recorded during follow-up. Multidisciplinary care included orthopedics, cardiology, pulmonology, and rehabilitation.
**Outcome and follow-up**	Alive at most recent follow-up (2026, age 5.5 years). Walking without support; cardiac valve disease progressive on serial echocardiograms; growth maintained at <3rd percentile.
**Interpretation**	ERT began in Q1 2021, before WES in 2023. This case illustrates complementary WGS after a single *GALNS* finding, but does not support treatment initiation attributed to WES.

Patient P2-06

**Demographics**	Male, born 2015; aged 8.9 years at genomic-test referral; mixed Taiwanese-European (Han Chinese maternal, European paternal).
**Clinical presentation**	Intellectual disability, autism spectrum disorder, behavioral disorder, rigid joints, hip dysplasia, verbal developmental delay, allergic rhinitis, ADHD since age 3, chronic diarrhea since infancy (3–4 episodes/day), bilateral genu valgum (progressive).
**Pre-sequencing workup**	Array CGH: within normal limits. Bilateral lower limb radiographs (external imaging, 2023): bilateral genu valgum with flattened femoral head epiphyses. Orthopaedic intervention (growth plate surgery) at an external tertiary center, 2023.
**Genomic findings and diagnostic interpretation**	The original registry lists WGS through KimForest in 2024, with a 28-day submission-to-report interval; the later summary calls the test singleton WES. *HGSNAT* c.607C>T, p.(Arg203Ter), is recorded; only one allele is documented. This does not establish a biallelic molecular diagnosis.
**Treatment and management**	Supportive symptomatic management was recorded. No disease-modifying treatment was documented in the available case summary.
**Outcome and follow-up**	Family relocation limited follow-up. MPS IIIC was recorded as the clinical diagnosis at age 8.9 yr, but its biochemical confirmation and second molecular allele are not documented adequately in this summary.
**Interpretation**	Bi-racial family with international residence — geographic relocation limits long-term follow-up.

Patient P2-07

**Demographics**	Male, born 2024; aged 0.4 years at genomic-test referral; Han Chinese (Taiwan); twin B; preterm 36+3 weeks.
**Clinical presentation**	Newborn screening-detected low IDS enzyme activity (1.0 µmol/g protein/4h, reference 12.89–131.83). Hepatomegaly (8.48 cm), ventricular dilatation, bilateral enlarged occipital horns, mild brain atrophy on MRI, abnormal MRS choline/NAA ratio, mild gibbus deformity emerging.
**Pre-sequencing workup**	NBS tandem mass + IDS enzyme assay (three sequential confirmatory tests). Urinary GAG biomarkers (DMB ratio, heparan sulfate, dermatan sulfate) markedly elevated. Brain ultrasound and MRI. Serial echocardiography. Targeted *IDS* gene analysis (no pathogenic mutation found on standard panel).
**Genomic findings and diagnostic interpretation**	The original registry lists WGS through KimForest in 2024, with a 43-day submission-to-report interval; the later summary calls the test singleton WES. The *IDS* c.1181-15C>A finding is recorded as hemizygous. The later summary labels it VUS with literature and biochemical support for MPS II.
**Treatment and management**	Enzyme replacement therapy (idursulfase) initiated Q1 2025 at an external medical center. Hematopoietic stem cell transplantation performed Q3 2025 at an external transplant center. Post-BMT short tandem repeat analysis (Q4 2025) confirmed donor chimerism.
**Outcome and follow-up**	Alive at most recent follow-up (2026). Walking since Q4 2025. The clinical summary records urinary heparan sulfate of 992.71, 7.74, and 35.91 micrograms/mL before treatment, after ERT, and after HSCT, respectively. IDS enzyme activity was recorded as increasing from 1.0 to 2.15; sampling dates and units were not specified.
**Interpretation**	ERT and HSCT with serial biomarker monitoring were recorded. These observations document the clinical course but cannot isolate the effects of either treatment or of genomic testing.

Patient P2-08

**Demographics**	Male, born 2022; aged 2.5 years at genomic-test referral; Han Chinese (Taiwan).
**Clinical presentation**	Multiple xanthomas over bilateral posterior knees and ankles (9-month history). Severe hypercholesterolemia (total cholesterol 482–501, LDL 416–439, triglycerides 98–330 mg/dL). Background congenital cardiac disease: pulmonary atresia with intact ventricular septum (PAIVS) s/p pulmonary valve perforation + PTPV at neonatal period; hypoplastic right ventricle; severe tricuspid regurgitation; home O_2 (SpO_2 85–95%). Parental cholesterol elevated (father LDL 196, mother LDL 153) consistent with heterozygous carriage.
**Pre-sequencing workup**	Standard lipid panel. Cardiac follow-up imaging (separate). No plant sterol assay available locally at time of presentation.
**Genomic findings and diagnostic interpretation**	The original registry records WES+Mt through KimForest in 2025 (27-day interval), with *ABCG5* c.1166G>A, p.(Arg389His), and c.64C>T, p.(Gln22Ter). The later summary assigns VUS and pathogenic labels, respectively, and additionally records hemizygous *FLNA* c.5479A>G, p.(Thr1827Ala), as VUS. *FLNA* is absent from the matching registry row. Parental Sanger testing was recommended; trans configuration and the *FLNA* contribution remain unconfirmed.
**Treatment and management**	Dietary counseling (low phytosterol diet: avoidance of plant oils, nuts, seeds, chocolate, olive oil, avocado). Pending ezetimibe consideration. Parental Sanger sequencing recommended.
**Outcome and follow-up**	Alive at most recent follow-up (2025, age 2.5 years). Recently diagnosed; long-term cardiovascular and dietary management ongoing. Follow-up shared with a regional medical center.
**Interpretation**	*ABCG5* and *FLNA* findings were counted under the inclusive rule. The *FLNA* VUS is not an established second diagnosis.

Patient P2-09

**Demographics**	Male, born 2006; aged 11.7 years at genomic-test referral; Han Chinese (Taiwan).
**Clinical presentation**	Psychomotor retardation and poor growth from infancy. Neonatal seizure at day 3 of life. Hypertelorism, deep-set eyes, flat nasal bridge, hypoplastic floppy cupped ears, loose skin, hyperextensible joints (mother also Beighton score 8/9), very thin muscle mass, short stature, oligodontia (24 teeth, dysplastic), urinary incontinence with bedwetting, behavioral challenges (biting), poor wound healing since 2021.
**Pre-sequencing workup**	Karyotype (external): 46,XY. Array CGH (external regional medical center): 7q duplication, maternally inherited. SMA screen: negative. Echocardiography (2017): within normal limits. Bone age (age 7): 6–7 years. Thyroid function WNL. PT/INR/APTT, CBC, full biochemistry all WNL.
**Genomic findings and diagnostic interpretation**	NHRI referral in 2018, described as trio WES in the later summary. The original registry lists *SLC6A9* p.(Leu28Pro) as "not found" and p.(Tyr133Phe) as VUS, whereas the later summary assigns LP to both. These conflicting labels require dated classification evidence. *ZNF469* p.(Pro1363Arg) and p.(Arg3090Ter) are recorded as likely benign and pathogenic, respectively; they are not counted as a biallelic second diagnosis. A maternally inherited 7q duplication was identified separately.
**Treatment and management**	Symptomatic management. Child psychiatry referral. PT since 6 months of age. Special education.
**Outcome and follow-up**	Alive at age 15 (2022 follow-up); special education transferred between schools with notable behavioral improvement; growth delay persistent; PMR stable.
**Interpretation**	*The SLC6A9-related diagnosis and both variant classifications require reconciliation. ZNF469 remains a carrier finding in this analysis; unresolved connective-tissue features do not by themselves establish a second diagnosis.*

Patient P2-10

**Demographics**	Male, born 2014; aged 4.5 years at genomic-test referral; Han Chinese (Taiwan).
**Clinical presentation**	Walking without support delayed (3y4m); slurred and delayed speech with echolalia; gross motor, endurance, cognition, memory all delayed; stereotypic hand movements; tension tremor; expressive speech disorder; intermittent strabismus; wide-spaced unstable gait with falls; head tilting; round back. Globally autistic features. No seizures.
**Pre-sequencing workup**	Brain MRI (external, 2018): suggestive of Joubert syndrome features (thin superior cerebellar peduncles); MRS unremarkable. Prior brain MRI (2015): WNL. Echocardiography (external, 2017): small PFO. NCV/EMG (2018): no peripheral neuropathy or myopathy. EEG (external, 2017): mild anomaly. EEG (external, 2015): r/o diffuse cortical dysfunction. Plasma carnitine WNL; lactate 21.7 (mildly elevated). Array CGH (2015): WNL. SMA screen: negative. Thyroid function WNL.
**Genomic findings and diagnostic interpretation**	NHRI referral in 2018. The original registry records *ZSWIM6* p.Ala161fs without a classification and *COG4* p.(Ser236Trp) as VUS. The later summary supplies *ZSWIM6* c.481dup, a pathogenic label, and de novo/trio claims as recorded in the later case summary. Palmer et al. [24] described recurrent p.Arg913Ter, not this recorded frameshift. The *COG4* finding does not establish Saul–Wilson syndrome. This case is retained only in the exploratory metabolic-related series.
**Treatment and management**	CoQ10 10 mg BID since age 3; vitamin B6 since 3+ years; piracetam 200 mg/mL 4 cc QD since 2018; amantadine 100 mg ¼ tablet BID (intermittent). PT/OT since 10 months, ST since 1 year, acupuncture since 2018. Deferred kindergarten entry.
**Outcome and follow-up**	Alive at most recent follow-up (2021, age 7). Cognitive and behavioral improvement reported with CoQ10 + vitamin B6 + piracetam; some expressive speech gains.
**Interpretation**	Metabolic eligibility is based on an uncertain *COG4* finding, which does not establish a glycosylation disorder.

Patient P2-11

**Demographics**	Male, born 2004; aged 14.8 years at genomic-test referral; Han Chinese (Taiwan).
**Clinical presentation**	Moderate intellectual disability with autism spectrum traits; behavioral problems; ADHD (combined type); developmental and behavioral challenges. Mild joint contracture. Past history: esotropia s/p surgery age 4; right pleural empyema s/p thoracoscopic decortication age 7; allergic rhinitis. Lives in a special-needs residential facility from Monday to Friday, returns to legal guardian (maternal aunt) at weekends.
**Pre-sequencing workup**	Karyotype (external, 2005): 46,XY .ish ins(2;6)(p15;q22.2q25), t(3;6;9)(p23;q25;q32), inv(7)(p15.3q31.2) — complex chromosomal rearrangement; parental karyotypes normal. Array CGH (2017): negative (incl. high-resolution SNP probes). Allergen panel: HDM class 3. CBC, CRP, biochemistry WNL.
**Genomic findings and diagnostic interpretation**	NHRI referral in 2019. The original registry labels *SLC6A8* p.(Thr178Ser) benign, whereas the later summary labels it LP. *PIEZO2* p.(Val1660Ala) is VUS and marked pending validation; no cDNA description is supplied. The registry contains no trio/singleton field, and the later trio claim conflicts with unavailable parental samples. These source differences remain unresolved.
**Treatment and management**	Symptomatic. Psychiatry: methylphenidate (Concerta 27 mg) + aripiprazole (Abilify 5 mg) for ADHD/behavior. Special education.
**Outcome and follow-up**	Alive at most recent follow-up (2025, age 20). Resides in special-needs facility; morning vocational placement, afternoon woodworking. Behavioural status improved after schooling and environment adjustment.
**Interpretation**	The *SLC6A8* classification differs between sources (benign versus LP). Recorded contractures and corrected esotropia do not establish *PIEZO2* pathogenicity.

Patient P2-12

**Demographics**	Male, born 1996; aged 23.1 years at genomic-test referral (pediatric-onset disease); Han Chinese (Taiwan).
**Clinical presentation**	Frequent generalized seizures and absence episodes since childhood; progressive motor deterioration; muscle weakness; squint with poor visual acuity; intermittent multiple joint swelling; easy fatigue; rapid visual field narrowing since 2020 (training with white cane). Pre-existing diagnoses: chronic diarrhea with PMR; retinitis pigmentosa; glaucoma.
**Pre-sequencing workup**	Lactate 8.7 mg/dL (normal range). Ammonia 33. CK normal. Coenzyme Q10 measurements at two external centers: 315 nM (reference 500–1970) and 0.41 µmol/L (reference 0.5–1.97) — both mildly decreased despite supplementation. Acid maltase activity (an external academic center, 2011): WNL (Pompe ruled out). Muscle biopsy (external, an external medical center): minimal change myopathy.
**Genomic findings and diagnostic interpretation**	NHRI referral in 2019, described as trio WES in the later summary. The original registry labels *SLC25A41* p.(Ala229Thr) likely benign and p.(Trp93Arg) VUS, whereas the later summary labels both LP. *SLC25A5* p.(Phe271Leu) and p.(Asn277Ser) are VUS in both sources. The registry lists the inheritance models as unknown. These remain candidate findings with unresolved classification and gene–disease evidence, not established dual mitochondrial diagnoses.
**Treatment and management**	CoQ10 (from a regional medical center), L-carnitine (from another external center), immune therapy (consulted from an external academic center). Pulse parenteral steroid every 3 months since 2016.
**Outcome and follow-up**	Alive at most recent follow-up (2020). Continuing visual decline; multiple medication regimens; long diagnostic odyssey of >20 years. Elder sister has separate hematological condition.
**Interpretation**	The findings involve candidate gene–disease associations; LP labels alone do not establish a clinical molecular diagnosis.

Patient P2-13

**Demographics**	Male, born 2018; aged 1.9 years at genomic-test referral; Han Chinese (Taiwan).
**Clinical presentation**	Motor delay (rolling 9 months; crawling 14 months; walking alone 19 months); speech delay (few words, only “mama”); microcephaly; autism with global delay; bruxism; self-injurious behavior (biting self/others, head-banging); pain insensitivity; chronic constipation; sleep disturbance; emotional instability; possible gelastic seizure episodes at age 3+; mild facial dysmorphism (coarse features, flat nasal bridge, hairy eyebrows, dysplastic ears).
**Pre-sequencing workup**	Array CGH: arr 4q23q24 (99,022,899–104,131,466) ×1.0 — 5.109 Mb interstitial deletion containing 25 OMIM genes. Brain MRI (2023): no significant abnormality. Bone age (age 1.9): ~18 months (delayed). 25-OH vitamin D 19.70 (deficient). EEG (2022, 2023): mildly abnormal. Renal echocardiography: right mild pelvic dilatation with small hyperechoic lesion.
**Genomic findings and diagnostic interpretation**	WES referral through Compass Bioinformatics in 2020; Jing-Ji is recorded separately in the aCGH field. The registry lists *NDUFA10* c.404T>C, p.(Leu135Ser), and c.1090_1110del, p.(Ser364_Arg370del). LP labels come from the later summary. The deletion remains inconsistent with the listed NM_004544.4 reference protein length of 355 amino acids. The 4q23–q24 deletion is a separate cytogenetic finding.
**Treatment and management**	Piracetam 1 cc BID; coenzyme Q10 10 mg BID, titrated to 50–100 mg/day. Occupational therapy ×2/week; physiotherapy and speech therapy ×1/week. Magnesium L-threonate trial.
**Outcome and follow-up**	Alive at the most recent documented follow-up (2023). The mother reported improvement in cognition, response, mood stability and eye contact after CoQ10 dose escalation. This uncontrolled observation does not establish a variant-specific treatment effect. The patient attended special-education kindergarten.
**Interpretation**	Co-existing cytogenetic (4q23-q24 deletion) and molecular (*NDUFA10*) findings; for P2 paper, *NDUFA10* is indexed and 4q deletion footnoted as parallel cytogenetic finding.

Note. Table S1 uses study identifiers only. Provider pathways are cross-referenced in Table S3. Inclusive multi-locus findings include unresolved additional findings; they do not all represent confirmed diagnoses. AD, autosomal dominant; AR, autosomal recessive; ERT, enzyme replacement therapy; HSCT, hematopoietic stem cell transplantation; IEM, inherited metabolic disorder; MPS, mucopolysaccharidosis; NBS, newborn screening; NDD, neurodevelopmental disorder; VUS, variant of uncertain significance; WES, whole-exome sequencing; WGS, whole-genome sequencing.

Table S2. Recorded descriptions of 27 variants across 18 gene-level findings

Tables S2A and S2B are linked by variant number, study identifier, and gene. Classifications and supporting evidence are recorded source annotations. The class column preserves later case-summary labels; six differences from the registry are stated in Table S2B. Database identifiers are included only where verified. Population-frequency, prediction, and ACMG evidence entries reproduce the case summaries and were not independently revalidated.

Table S2A. Variant identity and classification

Variant no.	Pt ID	Gene	Transcript	cDNA (HGVS)	Protein (HGVS)	Zygosity	Inh.	Later summary class
1	**P2-01**	*G6PD*	NM_001042351.3	c.1466G>T	p.(Arg489Leu)	Het (X-linked, F)	XLR	LP; registry conflict
2	**P2-02**	*GUSB*	NM_000181.4	c.104C>A	p.(Ser35Ter)	Het	AR	P
3	**P2-02**	*GUSB*	NM_000181.4	c.1454C>T	p.(Ser485Phe)	Het	AR	VUS
4	**P2-03**	*MMUT*	NM_000255.4	c.1106G>A	p.(Arg369His)	Het	AR	LP
5	**P2-03**	*MMUT*	NM_000255.4	c.1677-1G>A	p.?	Het	AR	LP
6	**P2-03**	*PTPN11*	NM_002834.5	c.794G>A	p.(Arg265Gln)	Het	AD	P
7	**P2-04**	*SLC37A4*	NM_001164277.2	c.1042_1043del (source coordinates; normalization unverified)	Source: p.Leu348fs*53; full consequence unverified	Het	AR	VUS
8	**P2-04**	*SLC37A4*	NM_001164277.2	c.898C>T	p.(Arg300Cys)	Het	AR	LP
9	**P2-05**	*GALNS*	NM_000512.5	c.953T>G	p.(Met318Arg)	Het	AR	P
10	**P2-05**	*GALNS*	NM_000512.5	c.423-862C>T	p.?	Het	AR	VUS
11	**P2-06**	*HGSNAT*	NM_152419.3	c.607C>T	p.(Arg203Ter)	Het	AR	P
12	**P2-07**	*IDS*	NM_000202.8	c.1181-15C>A	p.?	Hemi (M)	XLR	VUS
13	**P2-08**	*ABCG5*	NM_022436.3	c.64C>T	p.(Gln22Ter)	Het	AR	P
14	**P2-08**	*ABCG5*	NM_022436.3	c.1166G>A	p.(Arg389His)	Het	AR	VUS
15	**P2-08**	*FLNA*	NM_001110556.2	c.5479A>G	p.(Thr1827Ala)	Hemi (M)	XL	VUS

Variant no.	Pt ID	Gene	Transcript	cDNA (HGVS)	Protein (HGVS)	Zygosity	Inh.	Later summary class
16	**P2-09**	*SLC6A9*	NM_006934.4	c.83T>C	p.(Leu28Pro)	Het	AR	LP; registry conflict
17	**P2-09**	*SLC6A9*	NM_006934.4	c.398A>T	p.(Tyr133Phe)	Het	AR	LP; registry conflict
18	**P2-10**	*ZSWIM6*	NM_020928.2	c.481dup	p.?; source frameshift incomplete	Het	AD	P
19	**P2-10**	*COG4*	NM_015386.4	c.707C>G	p.(Ser236Trp)	Het	AD	VUS
20	**P2-11**	*SLC6A8*	NM_005629.4	c.532A>T	p.(Thr178Ser)	Hemi (M)	XLR	LP; registry conflict
21	**P2-11**	*PIEZO2*	NM_022068.2	Not recorded	p.(Val1660Ala)	Het	AD	VUS
22	**P2-12**	*SLC25A41*	NM_173637.4	c.685G>A	p.(Ala229Thr)	Het	AR	LP; registry conflict
23	**P2-12**	*SLC25A41*	NM_173637.4	c.277T>C	p.(Trp93Arg)	Het	AR	LP; registry conflict
24	**P2-12**	*SLC25A5*	NM_001152.5	c.811T>C	p.(Phe271Leu)	Hemi (M)	XL	VUS
25	**P2-12**	*SLC25A5*	NM_001152.5	c.830A>G	p.(Asn277Ser)	Hemi (M)	XL	VUS
26	**P2-13**	*NDUFA10*	NM_004544.4	c.404T>C	p.(Leu135Ser)	Het	AR	LP
27	**P2-13**	*NDUFA10*	NM_004544.4	Source: c.1090_1110del; reference mismatch	Source: p.(Ser364_Arg370del); reference mismatch	Het	AR	LP

Table S2B. Supporting evidence and annotations

Variant no.	Pt ID	Gene	ACMG criteria	ClinVar ID	gnomAD AF	Origin	In silico (SIFT/PROVEAN/CADD)	Notes / references
1	**P2-01**	*G6PD*	PS3, PM2, PP3	—	<0.001	Inherited	Damaging / Damaging / 28	Original registry: pathogenic; later summary: LP. Registry autosomal-dominant label is inconsistent with X-linked G6PD.
2	**P2-02**	*GUSB*	PVS1, PM2, PP3	—	Absent	Paternal	Nonsense / N/A / N/A	Pathogenic null allele; paternal inheritance confirmed by Sanger.
3	**P2-02**	*GUSB*	PM2, PP3, BS2-conflicting	—	<0.001	Maternal (loss of contact)	Damaging / Damaging / 28	VUS in the later summary, with reported trans configuration. The enzyme defect and phenotype support the clinical diagnosis; the variant remains uncertain.
4	**P2-03**	*MMUT*	PS3, PM2, PM5, PP3	VCV000203846	<0.001	Parental (not specified)	Damaging / Damaging / 32	Classical *MMUT* variant in mut(0) MMA; literature-supported.
5	**P2-03**	*MMUT*	PVS1, PM2, PP3	—	Absent	Parental (not specified)	Splice / N/A / 33	Canonical splice acceptor variant; predicted complete loss of function.
6	**P2-03**	*PTPN11*	PS1, PS3, PM1, PM2, PM6, PP3	VCV000040522	Absent	Presumed de novo; not confirmed	Damaging / Damaging / 28	Pathogenic label from the later clinical summary; de novo status is presumed.
7	**P2-04**	*SLC37A4*	PVS1, PM2, conflicting	—	Absent	Maternal	Frameshift / N/A / N/A	Frameshift variant; predicted loss of function. VUS due to limited literature.
8	**P2-04**	*SLC37A4*	PS3, PM2, PP3	—	<0.001	Paternal	Damaging / Damaging / 30	Missense substitution at conserved residue; published in GSD Ib cases.
9	**P2-05**	*GALNS*	PS3, PM1, PM2, PP3, PP5	—	Absent	Paternal	Damaging / Damaging / 32	Established pathogenic variant in MPS IVA; literature-supported.
10	**P2-05**	*GALNS*	PM2, PP3	—	<0.001	Maternal	SpliceAI 0.45 / N/A / N/A	Identified by WGS only (missed by WES); deep-intronic variant predicted to affect splicing.
11	**P2-06**	*HGSNAT*	PVS1, PS3, PM2	—	Absent	Maternal	Nonsense / N/A / N/A	Only one *HGSNAT* allele is documented; the pathogenic label comes from the later summary. A biallelic molecular diagnosis is not established.

Variant no.	Pt ID	Gene	ACMG criteria	ClinVar ID	gnomAD AF	Origin	In silico (SIFT/PROVEAN/CADD)	Notes / references
12	**P2-07**	*IDS*	PM2, PP3, PS3-supporting	—	Absent	Maternal (presumed)	SpliceAI 0.62 / N/A / N/A	VUS in the later summary. Biochemical findings support the recorded MPS II diagnosis but do not alone establish variant pathogenicity.
13	**P2-08**	*ABCG5*	PVS1, PS3, PM2	—	Absent	Paternal (presumed)	Nonsense / N/A / N/A	Established sitosterolemia variant (Lipids Health Dis 2022;21:11).
14	**P2-08**	*ABCG5*	PM2, conflicting	—	<0.001	Maternal (presumed)	Tolerated / Neutral / 22	*ABCG5* VUS in a patient assessed for sitosterolemia. Parental testing was recommended; trans configuration is not established in the available documentation.
15	**P2-08**	*FLNA*	PM2, PP3	Not reported	Absent	Maternal (presumed)	Damaging / Damaging / 27	Not listed in matching original registry row. Later summary records *FLNA* VUS; segregation and cardiac contribution remain uncertain.
16	**P2-09**	*SLC6A9*	PS3, PM1, PM2, PP3	—	Absent	Parental (not specified)	Damaging / Damaging / 28	Original registry: “not found”; later summary: LP. Original classifier and dated evidence required.
17	**P2-09**	*SLC6A9*	PS3, PM2, PP3	—	Absent	Parental (not specified)	Damaging / Damaging / 27	Original registry: VUS; later summary: LP. Same gene as variant 16; not a second locus.
18	**P2-10**	*ZSWIM6*	PVS1, PS2, PM2, PP3, PP5	—	Absent	Reported de novo in later case summary	Frameshift / N/A / N/A	Registry: p.Ala161fs, no classification. Later summary: c.481dup, pathogenic/de novo. The full protein consequence is incomplete; Palmer et al. [24] describe p.Arg913Ter, a different variant.
19	**P2-10**	*COG4*	PM2, PP3	—	Absent	Inherited (presumed)	Damaging / Damaging / 28	*COG4* VUS; Saul–Wilson syndrome not established. The recurrent p.Gly516Arg disease mechanism cannot be assumed for p.(Ser236Trp).
20	**P2-11**	*SLC6A8*	PS3, PM2, PP3	—	Absent	Maternal (presumed, parents deceased)	Damaging / Damaging / 27	Original registry: benign; later summary: LP. Do not assume a validated reclassification.
21	**P2-11**	*PIEZO2*	Not recorded	Not recorded	Not recorded	Unknown (both parents deceased; segregation not testable)	Not recorded	Additional *PIEZO2* VUS. Contractures, intellectual disability and corrected esotropia were recorded, but a causal contribution was not established. The original registry supplies only the protein change and marks validation as pending; cDNA and segregation evidence are unavailable.

Variant no.	Pt ID	Gene	ACMG criteria	ClinVar ID	gnomAD AF	Origin	In silico (SIFT/PROVEAN/CADD)	Notes / references
22	**P2-12**	*SLC25A41*	PS3, PM2, PP3	—	Absent	Paternal (presumed)	Damaging / Damaging / 28	Original registry: likely benign; later summary: LP. Candidate association remains unresolved.
23	**P2-12**	*SLC25A41*	PS3, PM2, PP3	—	Absent	Maternal (presumed)	Damaging / Damaging / 29	Original registry: VUS; later summary: LP. Candidate association and source phase remain unresolved.
24	**P2-12**	*SLC25A5*	PM2, PP3	Not reported	Absent	Maternal (presumed)	Damaging / Damaging / 26	Candidate *SLC25A5* finding; no established second mitochondrial diagnosis.
25	**P2-12**	*SLC25A5*	PM2, PP3	Not reported	Absent	Maternal (presumed)	Damaging / Damaging / 27	*Second hemizygous SLC25A5 variant in the same male; not compound heterozygosity.*
26	**P2-13**	*NDUFA10*	PS3, PM2, PP3	—	Absent	Parental (not specified)	Damaging / Damaging / 28	Mitochondrial complex I deficiency.
27	**P2-13**	*NDUFA10*	PM2, PM4, PP3	—	Absent	Parental (not specified)	In-frame deletion / N/A / N/A	The reported deletion/protein coordinates are inconsistent with the coding length of NM_004544.4; the source description is retained and flagged in Table S2A.

Note. Predicted protein consequences are enclosed in parentheses; p.? indicates an undetermined consequence. Transcript accession and version are shown in Table S2A. A cDNA description was not reconstructed when it was absent or inconsistent in the source. Phase is not assumed from the presence of two heterozygous variants. Source classifications are retained for traceability, including unresolved candidate-gene findings; they are not newly validated classifications. Database identifiers were corrected where an exact match was verified. Numerical prediction scores and allele frequencies are reproduced from the case summaries as described above. A dash indicates that no verified database identifier is supplied. P2-09 also had *ZNF469* p.(Arg3090Ter) and p.(Pro1363Arg), recorded as pathogenic and likely benign, respectively; these carrier findings are outside the 27-variant summary. P, pathogenic; LP, likely pathogenic; VUS, variant of uncertain significance; AD, autosomal dominant; AR, autosomal recessive; XL, X-linked; XLR, X-linked recessive.

Figure S1. Disease groups used for the exploratory analysis

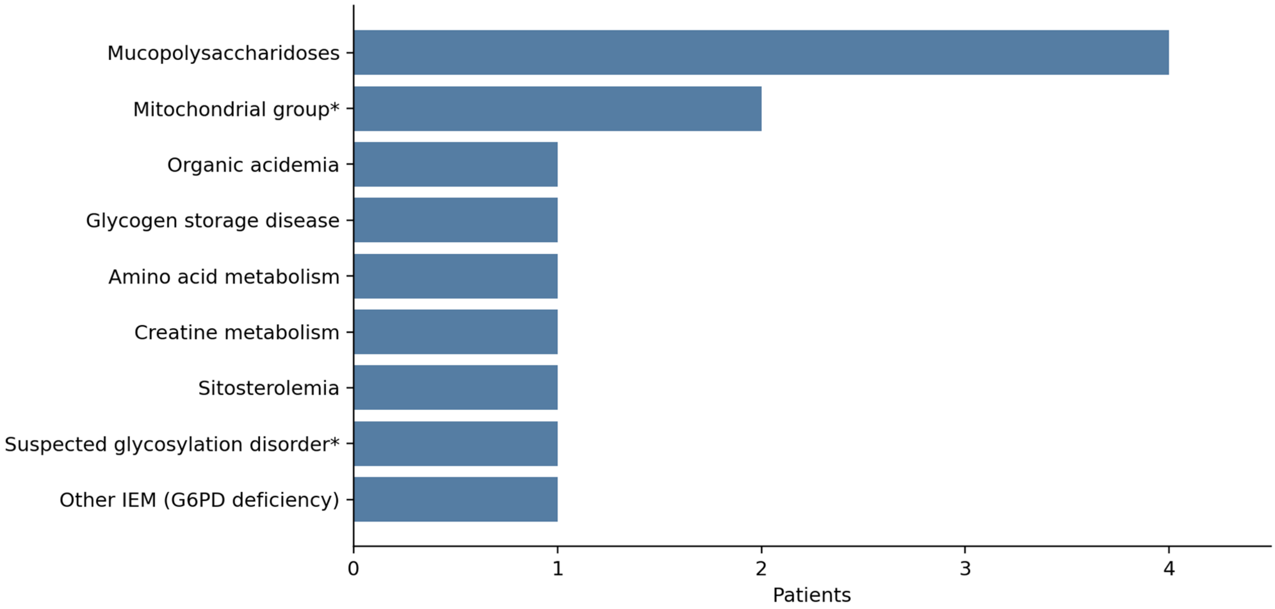

Counts refer to 13 patients. Mucopolysaccharidoses form the largest group. The mitochondrial group includes P2-12 with candidate gene findings, and the glycosylation group reflects the *COG4* VUS in P2-10. These grouping labels do not establish confirmed diagnoses. The asterisks identify the mitochondrial and glycosylation groups whose diagnostic uncertainty is described here.

Figure S2. Age at genomic-test referral by patient

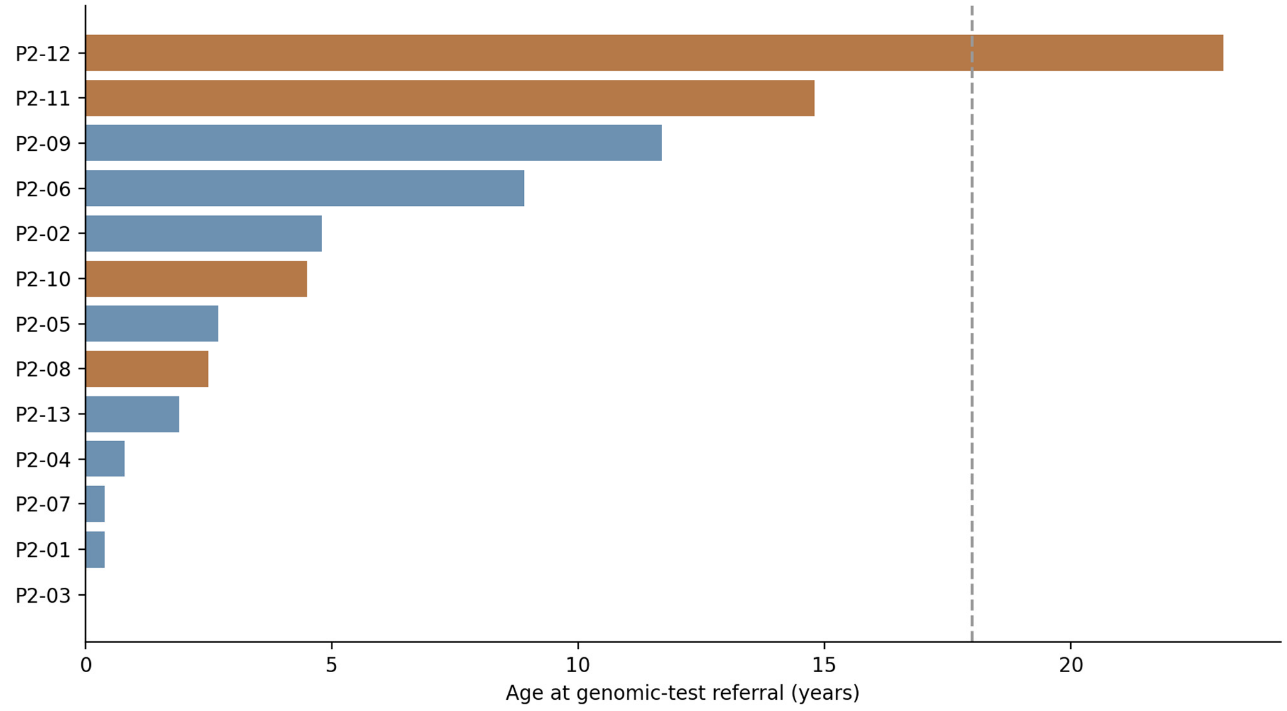

Age at genomic-test referral by patient. Brown bars mark inclusive multi-locus findings, including VUSs and candidate associations; blue bars mark the remaining cases. The dashed line denotes 18 years. P2-03 was referred on day 2 of life (rounded to 0.0 yr). Referral age does not measure diagnostic delay.

Table S3. Recorded provider pathways and technical-information availability

Code	Patients and period	Provider by test field	Modality and source limits
NHRI	P2-01, 09, 10, 11, 12; 2018–2019	NHRI program; no individual sequencing laboratory specified	Four trios and one singleton in later summaries; P2-11 family structure uncertain
A	P2-06, 07, 08; 2024–2025	KimForest	P2-06 and P2-07: WGS; P2-08: WES+Mt
B	P2-03, 04, 05; 2022–2023	P2-03 WES: Compass Bioinformatics; Sanger: Ao-Ji. P2-04 and P2-05 sequencing: Ao-Ji	P2-05: research funding, no charge; complementary WGS in later summary
C	P2-02, 13; 2020	WES: Compass Bioinformatics for both. P2-13 aCGH: Jing-Ji	Sequencing and aCGH providers recorded separately

Table S3 lists providers by test type using the registry fields. Codes A–C link to earlier versions and do not identify uniform sequencing laboratories. Technical methods are described in Section 2.3. The four dated genomic-testing episodes were 117, 28, 43, and 27 days; P2-06 and P2-07 are recorded as WGS. Ao-Ji and Jing-Ji are romanized registry provider labels; KimForest and Compass Bioinformatics are the English provider names.
